# Design and Application of Nanozymes: Current Trends and Cutting‐Edge Breakthroughs

**DOI:** 10.1002/advs.76811

**Published:** 2026-07-30

**Authors:** Kelong Fan, Hui Wei, Xiyun Yan

**Affiliations:** ^1^ State Key Laboratory of Biomacromolecules, Institute of Biophysics Chinese Academy of Sciences Beijing China; ^2^ Nanozyme Laboratory in Zhongyuan Henan Academy of Innovations in Medical Science Zhengzhou Henan China; ^3^ School of Biomedical Engineering, State Key Laboratory of Analytical Chemistry for Life Science, Chemistry and Biomedicine Innovation Center (ChemBIC) Nanjing University Nanjing Jiangsu China

As a type of material that integrates the physicochemical advantages of nanomaterials with enzyme‐mimicking catalytic functions, nanozymes have demonstrated significant potential because of their ability to bridge multiple disciplines, such as physics, chemistry, biology, and medicine. Following nearly two decades of development, through the efforts of researchers across various fields, nanozymes have evolved into a research field characterized by both breadth and depth. Moreover, in recent years, the design and application of nanozymes have consistently achieved numerous remarkable breakthroughs.

This special issue of *Advanced Science* encompasses 40 papers contributed by researchers across the globe. These studies span a diverse range of fields, including the optimization of the catalytic performance of nanozymes, environmental conservation, biosensing, antioxidant therapy, immunomodulatory therapy, antibacterial therapy, and other innovative biomedical applications.

Significant progress has been achieved in enhancing the catalytic activity of nanozymes and simultaneously improving their stimuli‐responsiveness and stability. Li and co‐workers (article number 202519424) systematically investigated the role of ligand regulation in carbon dot‐Fe nanozymes and demonstrated that ligand identity strongly influences both peroxidase (POD)‐like and UV‐induced oxidase‐like activities while also revealing a synergistic UV‐enhanced catalytic mechanism that significantly improves reaction kinetics. These findings highlight the importance of tailoring ligand‐metal interactions to modulate nanozyme activity and provide a foundation for the development of next‐generation catalytic nanomaterials. Based on the concept of ligand‐directed nanozyme engineering, Chen and co‐workers (article number 202519268) employed renewable lignin as a sustainable ligand scaffold to construct a Ce‐doped hydrolase nanozyme with remarkable catalytic stability and efficiency. Their lignin‐based nanozymes exhibited enhanced hydrolytic activity under elevated temperatures and effectively disrupted bacterial biofilms, demonstrating how rational ligand design can simultaneously improve catalytic performance, environmental sustainability, and practical applicability. To increase the mechanical robustness of nanozymes and facilitate scalable fabrication, Bhargava and co‐workers (article number 202524355) designed an iron‐based metal‐organic framework (Fe‐BTC) directly grown on a 3D‐printed Ti‐Al‐V substrate, with phosphomolybdic acid incorporated in situ. Compared with conventional powder nanozymes, this integrated nanozyme platform demonstrated enhanced catalytic activity, superior stability, and excellent reusability, facilitating sensitive and reliable glucose sensing. Collectively, these studies underscore the critical role of coordination environments and ligand engineering in advancing nanozyme functionality and offer valuable strategies for the development of highly active, robust, and sustainable nanozyme systems.

Nanozymes have emerged as promising catalytic materials for addressing challenges in both environmental sustainability and energy conversion. Building upon these advances, Wu and co‐workers (article number 202519278) introduced a self‐powered CeO_2_/g‐C_3_N_4_ piezo‐responsive nanozyme platform capable of harvesting low‐frequency mechanical energy from ocean waves to drive enzymatic cascade reactions, thereby achieving efficient and environmentally benign marine antifouling without external energy input. To further extend the application scope of nanozymes, Zhang and co‐workers (article number 202519235) developed a Lewis pair‐engineered defective Cu‐doped Mn oxide nanozyme with enhanced hydrolytic and oxidative activities, enabling the efficient degradation of lignocellulosic biomass under mild conditions. Moreover, Dong and co‐workers (article number 202519402) comprehensively reviewed the development of nanozymes for energy and environmental applications, emphasizing their advantages in pollutant degradation, wastewater treatment, heavy metal detoxification, and antibacterial disinfection.

Recent advances in nanozyme‐based biosensing technologies have significantly expanded the capabilities of point‐of‐care testing. Stevens and co‐workers (article number 202523192) demonstrated the practical utility of platinum nanozyme‐enhanced lateral flow immunoassays (LFIAs) for ovarian germ cell tumor monitoring, combining optimized nanozyme‐antibody conjugation with advanced analytical modeling to achieve clinically relevant diagnostic performance. With respect to the need for higher sensitivity in LFIA platforms, Xiao and co‐workers (article number 202519130) developed a two‐stage nanozyme cascade amplification strategy based on Zn/WOX@Au@Pt nanozymes, achieving substantial signal enhancement and ultra‐sensitive detection of *Clostridium difficile* toxin B. Beyond individual sensing platforms, Chang and co‐workers (article number 202519136) provided a comprehensive overview of nanozyme‐integrated biochip systems, highlighting the synergistic integration of nanozyme‐catalyzed signal amplification, biochip‐based signal transduction, and artificial intelligence (AI)‐assisted data processing for intelligent detection applications. Complementing this perspective, Xia and co‐workers (article number 202524259) reviewed the emerging role of POD‐mimicking nanozymes in infectious disease diagnostics, emphasizing how rational nanozyme design, advanced biosensing architectures, and AI‐enabled optimization collectively contribute to the development of rapid, accurate, and accessible diagnostic technologies. Together, these studies illustrate the evolution of nanozyme‐enabled detection systems from assay optimization and signal amplification toward highly integrated, intelligent, and clinically translatable diagnostic platforms.

The regulation of oxidative stress has emerged as a central therapeutic strategy for a wide range of inflammatory and degenerative diseases. To provide a multifaceted therapeutic approach for inflammatory bowel disease (IBD), Zhang and co‐workers (article number 202512567) prepared *Dendrobium officinale*‐derived carbon dot nanozymes that scavenge reactive oxygen species (ROS), thereby restoring immune homeostasis and intestinal barrier integrity. In addition, Ding and co‐workers (article number 202514217) developed selenomethionine‐derived carbon dot nanozymes that efficiently attenuate intervertebral disc degeneration by eliminating excessive ROS and improving the local prooxidant microenvironment. Moreover, Xue and co‐workers (article number 202518191) reported iron oxide nanoparticles with ultra‐small size and excellent antioxidant enzyme‐like activities that reduce nitric oxide‐mediated neuroinflammation and promote microglial M2 polarization, ultimately improving recovery after ischemic stroke. Similarly, Wang and co‐workers (article number 202520714) engineered a microglial membrane‐coated PtRhIr/Ru single‐atom nanozyme capable of scavenging ROS and interrupting inflammatory cascades following intracerebral hemorrhage, thereby enhancing neurological repair. Apart from neurological diseases, Wu and co‐workers (article number 202521864) introduced myricetin‐arginine nanozymes that selectively target inflammatory macrophages to suppress both oxidative stress and osteoclast‐mediated bone destruction in rheumatoid arthritis. Moving toward infection‐associated inflammatory disorders, Wang and co‐workers (article number 202513536) developed 4‐mercaptobenzoic acid‐modified copper nanozymes that combined potent antibacterial activity with toxin neutralization and ROS regulation, effectively treating burn‐associated acute lung injury. In addition to controlling oxidative stress, emerging nanozyme systems have been designed to restore intracellular energy metabolism. Ning and co‐workers (article number 202523931) reported that polyvinylpyrrolidone‐modified palladium nanozymes not only scavenge ROS via superoxide dismutase (SOD)‐like activity but also mimic cytochrome *c* oxidase activity to improve mitochondrial function and alleviate aging‐related neurodegeneration. The therapeutic versatility of nanozymes is further illustrated by the work of Chen and co‐workers (article number 202517770), who developed a Mo‐N‐coordinated nanozyme capable of dynamically balancing oxidative reactions. This strategy not only improves the periodontal microenvironment but also promotes the restoration of the commensal microbiota and the regeneration of periodontal tissues. Interestingly, while most studies focus on eliminating excessive ROS, Qu and co‐workers (article number 202519561) proposed a contrasting yet complementary strategy involving a biomimetic polyphenol‐amino acid nanozyme that precisely generates controlled levels of H_2_O_2_ to harness beneficial oxidative eustress and stimulate tissue regeneration, providing a non‐pharmacological approach to alopecia therapy. In summary, these studies demonstrate the rapid evolution of antioxidant nanozyme research from simple ROS scavengers toward highly sophisticated platforms capable of simultaneously regulating oxidative stress, immune responses, metabolism, microbial ecosystems, and tissue regeneration, thereby opening new avenues for precision nanomedicine.

The catalytic activity of nanozymes and their distinctive physicochemical properties are also extensively utilized in the field of immunomodulatory therapy. Liu and co‐workers (article number 202515097) developed a T‐cell membrane‐coated MgCO_3_/Fe‐CD hybrid nanozyme that simultaneously reprograms tumor and T‐cell metabolism, amplifies radiotherapy‐induced oxidative stress, and relieves immune checkpoint suppression, thereby significantly enhancing antitumor immune responses. Another strategy proposed by Li and co‐workers (article number 202519186) exploited innate immunity to amplify immune responses. An iridium‐based nanozyme coated with the tumor cell lysate‐simulated dendritic cell membrane was engineered to target tumor cells and induce ferroptosis and pyroptosis through ROS generation and glutathione depletion. The oxidative damage‐induced release of mitochondrial DNA subsequently activated the cyclic GMP‐AMP synthase‐simulator of interferon gene (cGAS‐STING) pathway and elicited systemic antitumor immunity. Likewise, Zhao and co‐workers (article number 202519300) constructed Mn─Pt bimetallic nanozymes that synergistically amplified oxidative stress and activated the cGAS‐STING immune pathway via the release of Mn^2+^, resulting in enhanced radio‐immunotherapeutic efficacy against both primary and metastatic tumors. In addition to activating innate immunity, emerging nanozyme systems have been designed to trigger adaptive immune responses. Yang and co‐workers (article number 202512470) reported MgO_2_‐CuO_2_ bimetallic peroxide nanocomposites that produced excessive amounts of ROS to induce cuproptosis and pyroptosis, thereby promoting CD8^+^ T‐cell activation to induce an antitumor immune response. In addition to the treatment of established tumors, nanozyme‐based immunotherapy has been applied to prevent postoperative recurrence. In this context, Zhao and co‐workers (article number 202519431) developed a MnO_x_ nanozyme‐functionalized implantable scaffold that creates a sustained local immune niche through ROS‐mediated immunogenic cell death, hypoxia relief, and Mn^2+^‐driven immune activation. Apart from immune stimulation, how to overcome immune resistance in the tumor microenvironment remains another major challenge. To address this issue, Zhang and co‐workers (article number 202512575) designed Fe‐Cu MOF nanozymes capable of simultaneously inducing oxidative stress and inhibiting autophagy. This approach not only eradicated tumor cells but also restored the expression of MHC‐I, thus impeding the immune escape capacity of tumor cells. Furthermore, nanozymes also possess the potential to be utilized as vaccines for the prevention of immune‐related diseases. A dendritic cell membrane‐coated organic nanozyme was developed by Song and co‐workers (article number 202518037), which integrated photodynamic activity and catalytic ROS generation. Upon near‐infrared irradiation, this nanozyme could function as a tumor vaccine by activating T cells, leading to long‐term prevention of tumor occurrence. In addition to their application in tumor therapy, nanozymes can also serve as immunological adjuvants for the treatment of IBD. Gu and co‐workers (article number 202522027) utilized the POD‐like activity of Prussian blue nanozymes under acidic conditions to enable localized modulation of H_2_O_2_‐ and ROS‐enriched inflammatory cues and remodeling of the antigen‐processing microenvironment. In combination with antigenic epitope‐conjugated ferritin nanocarriers, Prussian blue nanozymes exhibit an excellent ability to induce durable humoral immunity for long‐term IBD therapy. In conclusion, the above studies highlight the enormous potential of multifunctional nanozymes for precise regulation of the immune response via various pathways.

The development of nanozyme‐based antimicrobial strategies has emerged as a promising approach for overcoming the limitations of conventional antibiotics in treating persistent and drug‐resistant bacterial infections. Lin and co‐workers (article number 202519334) introduced piezoelectric SnSe nanozymes that disrupt bacterial redox homeostasis through oscillatory redox stress, simultaneously enhancing host immune responses and achieving effective eradication of implant‐associated biofilms. Building upon the concept of exploiting bacterial metabolic vulnerabilities, Wu and co‐workers (article number 202520705) developed a Cu‐ZIF8‐Ru nanozyme with an asymmetric Cu─N─Ru catalytic center that triggers cuproptosis‐like bacterial death through enhanced multienzyme‐like catalysis and intracellular copper accumulation, providing a highly effective treatment strategy for multidrug‐resistant urinary tract infections. Expanding beyond direct bactericidal mechanisms, Li and co‐workers (article number 202513863) designed a bimetallic phenolic framework nanozyme (Que‐Fe‐CeMPF) that integrates ROS generation, biofilm matrix degradation, and immune modulation to simultaneously eliminate pathogens and promote tissue repair. Overall, these studies demonstrate the growing versatility of nanozyme‐based antimicrobial platforms, highlighting how the integration of catalytic regulation, programmed bacterial death pathways, biofilm disruption, and host immune modulation can provide powerful non‐antibiotic solutions for combating complex and drug‐resistant infections.

Cascade catalytic systems have emerged as powerful strategies for enhancing catalytic efficiency and expanding the therapeutic potential of nanozymes in biomedical applications. Muhammad and co‐workers (article number 202519393) developed mitochondria‐targeted silver single‐atom carbon‐dot nanozymes that integrate SOD‐ and glutathione peroxidase (GPx)‐like activities, as well as fluorescence imaging capabilities, enabling precise ROS scavenging and effective treatment of acute kidney injury. Extending the application of cascade nanozymes to neurological disorders, Gao and co‐workers (article number 202519340) engineered a Ru@Mn‐ZIF composite nanozyme capable of efficiently eliminating excessive ROS through cascade catalysis via SOD‐like and catalase (CAT)‐like activities, thereby mitigating neuroinflammation and secondary brain injury following intracerebral hemorrhage. Except for ROS elimination in inflammatory diseases, a prooxidant nanozyme cascade system (named Ru‐GOx‐PNN) was designed by Jiang and co‐workers (article number 202517528) for tumor therapy. By enveloping natural glucose oxidase (GOx), this nanozyme demonstrates a CAT‐like catalytic ability and formed a cascade reaction with GOx to continuously consume glucose at the tumor site. Moreover, the POD‐like activity of the nanozyme utilized the continuously generated H_2_O_2_ to produce hydroxyl radicals to kill tumor cells, ultimately attaining efficient and precise treatment of esophageal squamous cell carcinoma. Furthermore, Tang and co‐workers (article number 202519656) comprehensively reviewed the development of all‐nanozyme cascade reaction systems, summarizing their mechanistic foundations, structural design strategies, and emerging applications in biosensing and therapy. In summary, the aforementioned work highlights the substantial role of cascade catalysis in broadening the application prospects of nanozymes.

Notably, researchers have also investigated the potential of nanozymes to achieve disease treatment through the exertion of certain non‐classical biological functions or several unique components. Chen and co‐workers (article number 202524313) developed a hydrogen‐doped RhPd alloy nanozyme that integrates POD‐like activity with thermally triggered hydrogen gas release, establishing a metabolically amplified therapeutic strategy capable of sustaining intracellular ROS generation and enhancing antitumor immunity. In order to mitigate the severe adverse effects of chemotherapy on the heart, a bioinspired peroxisome‐engineered probiotic platform was prepared by Chen and co‐workers (article number 202519344), which combines antioxidant nanozymes with living probiotics to restore gut microbial homeostasis and alleviate chemotherapy‐induced cardiotoxicity through regulation of the gut‐heart axis. Building upon redox modulation, nanozymes can be designed to further regulate the functions of some critical proteins. For instance, Xi and co‐workers (article number 202512186) reported copper‐paeonol nanozymes that simultaneously induce ROS‐mediated tumor destruction and disrupt the actin‐bundling activity of fascin (a protein promoting tumor migration and invasion). Therefore, the fascin‐dependent metastasis and glycolytic reprogramming were effectively inhibited, providing a dual‐functional strategy against highly invasive cancers. As nanozyme functions become increasingly complex, precise delivery and spatiotemporal control have emerged as critical challenges. In light of this, Koo and co‐workers (article number 202523365) reviewed the emerging field of nanozyme microrobots, highlighting how the integration of catalytic nanomaterials with programmable robotic systems enables highly localized and stimulus‐responsive therapeutic interventions in otherwise inaccessible biological environments. Looking toward the substantial potential of layered double hydroxide (LDH)‐based materials to expand the versatility of nanozyme platforms, Guan and co‐workers (article number 202519115) comprehensively summarized LDH‐based nanozymes and emphasized their programmable multi‐active‐site architectures for precisely regulating pathological redox homeostasis and inflammatory responses. Additionally, since the report in 2021, cluster‐based nanozymes, as an innovative concept referred to as programmable nanozymes constructed from an atomically precise metal cluster, have offered a highly designable and efficient platform for biomedical applications. Given this, Zhang and co‐workers (article number 202519438) conducted a systematic review on the advancements of cluster‐based nanozymes and outlined their prospects in biomedicine. In a word, these studies illustrate the rapid evolution of nanozymes toward highly multifunctional and programmable biomedical platforms, paving the way for advanced diagnostics and precision therapeutics.

In summary, this special issue presents the most recent advancements within the realm of nanozymes, emphasizing both fundamental discoveries and applicational insights that are propelling this rapidly evolving field to the next phase. From the aforementioned advancements, we can forecast the future development prospects of nanozymes: (1) The catalytic performance of nanozymes (including catalytic activity, stability, and reusability) will witness continuous breakthroughs; (2) Nanozymes applied in biosensing and biomedicine will exhibit increasingly integrated functions. On the other hand, it is noteworthy that nanozymes still encounter challenges regarding clinical translation and the establishment of standardized evaluation systems, which require extended efforts of researchers from various fields.

Finally, we express our sincere appreciation to all the authors and reviewers who have made contributions. It is their valuable commitment and professional expertise that have resulted in the successful completion of this special issue.

